# Efficient Organic/Inorganic Hybrid Solar Cell Integrating Polymer Nanowires and Inorganic Nanotetrapods

**DOI:** 10.1186/s11671-016-1795-9

**Published:** 2017-01-05

**Authors:** Weizhe Xu, Furui Tan, Xiansheng Liu, Weifeng Zhang, Shengchun Qu, Zhijie Wang, Zhanguo Wang

**Affiliations:** 1 Key Laboratory of Photovoltaic Technique, Department of Physics and Electronics, Henan University, Kaifeng, 475004 China; 2 Key Laboratory of Semiconductor Materials Science, Institute of Semiconductors, Chinese Academy of Sciences, Beijing, 100083 China

**Keywords:** Nanowire, Nanotetrapod, Hybrid, Solar cells

## Abstract

Constructing a highly efficient bulk-heterojunction is of critical importance to the hybrid organic/inorganic solar cells. Here in this work, we introduce a novel hybrid architecture containing P3HT nanowire and CdSe nanotetrapod as bicontinuous charge channels for holes and electrons, respectively. Compared to the traditionally applied P3HT molecules, the well crystallized P3HT nanowires qualify an enhanced light absorption at the long wavelength as well as strengthened charge carrier transport in the hybrid active layer. Accordingly, based on efficient dissociation of photogenerated excitons, the interpercolation of these two nano-building blocks allows a photovoltaic conversion efficiency of 1.7% in the hybrid solar cell, up to 42% enhancement compared to the reference solar cell with traditional P3HT molecules as electron donor. Our work provides a promising hybrid structure for efficient organic/inorganic bulk-heterojunction solar cells.

## Background

Combining the potential advantage of organic semiconductor and inorganic nanocrystals, organic/inorganic hybrid bulk-heterojunction solar cell (HBSCs), one of the third generation thin-film solar cells, has attracted intense research efforts due to its unique advantage such as lightweight, low cost, good flexibility with large area, and easily tuned energy level alignment and so on. Based on extensive research efforts, the photovoltaic performance of HBSCs has been greatly improved. A power conversion of 5.5% was obtained in a hybrid containing PbS_x_Se_1-x_ nanocrystals and a low-bandgap polymer [1].

Compared to the traditional inorganic photovoltaic semiconductors, the hybrid composites in the HBSCs have relatively low charge mobility due to the disordered orientation of organic semiconductor molecules as well as discontinuously dispersed inorganic nanocrystals [2–4], confining further improvement of photovoltaic performance of HBSCs. Regarding this problem, many efforts were taken to optimize the charge transport efficiency in the HBSCs, such as annealing of the as-prepared hybrid films [5], modification on structure of molecule chains [6–9], and solvent treatment [10, 11]. Most of the process aimed at enhancing orientation of molecule chains or crystallization of polymer matrix so that to increase the charge motility. Likewise, direct self-assembly of polymer molecules to form organic nanowires (NWs) (such as poly(3-hexylthiophene (P3HT))) was also proved to be helpful in achieving an enhanced charge transport as well as highly efficient organic hybrid solar cells [12, 13].

Meanwhile, to obtain an optimized phase separation in the organic/inorganic hybrids, the inorganic electron acceptor was usually prepared with good monodispersion, at the cost of high charge mobility in the bulk materials. Thus, modulation on nanoparticels’ morphology was extensively investigated to increase electron transport and collection in the HBSCs, such as nanorods and nanotubes [14–16], orientated nanoarrays [17, 18] and core-shell-shaped nanocrystals [19–21], and so on. Monodispersed nanotetrapod (NT) was also a promising candidate because of its superiority in transporting electrons through an extended charge path with three-dimensional continuity [21–23].

Thus to HBSCs, the active layer with simultaneously efficient transportation of holes and electrons is of great importance. Although organic nanowires and inorganic nanotetrapods are attractive in charge transport, there is no reported work on the HBSCs simultaneously containing these two structures. Here in this work, we have fabricated this novel organic/inorganic HBSC composed of the above two efficient nano-building blocks: P3HT NWs and CdSe NTs as donor and acceptor, respectively. The active layer enables an efficient charge transport and collection through bicontinuous pathways for holes and electrons, which is highly favored by efficient HBSCs.

## Methods

### Synthesis of P3HT Nanowires, CdSe Nanotetrapods, and their Hybrid

The P3HT NWs were synthesized through a self-assembly method. Typically, P3HT polymer (Sigma-Aldrich, regioregular, average M_n_ 54,000–75,000) was dissolved in dichlorobenzene (DCB) and heated at 80–90 °C until a clear solution was obtained. Then, the solution was filtered through a 0.45-μm filter and allowed to be slowly cooled to 5–10 °C in a day without any agitation. The solution was kept at this temperature until a purple gel was obtained, indicating the formation of P3HT NWs. This sample was kept at low temperature before it was diluted with DCB for use.

CdSe NTs were synthesized according to the procedure in a literature [24]. Briefly, CdO (1 mmol), oleic acid (OA, 6 mmol), and 20 ml octadecene (ODE) were pumped at 140 °C under N_2_ flow for 30 min. After that, the temperature was raised to 260 °C at which a TOP-Se-CTAB (TOP: tri-n-octylphosphine, CTAB: Hexadecyl trimethyl ammonium Bromide) solution (containing 1 ml TOP, 0.5 mmol Se, 0.05 mmol CTAB, and 3 ml toluene) was injected quickly. The reaction was allowed and persisted for 5 min to grow the CdSe NTs. After the solution was cooled to room temperature, 10 ml acetone was injected and the red precipitation was collected by centrifugation at 4500 rpm. The obtained CdSe NTs were finally dissolved in DCB with desired concentration. The P3HT NWs:CdSe NTs hybrid solution was prepared by blending P3HT NWs and CdSe NTs DCB solution with different mass ratio and then stirring for at least 6 h. Reference sample was prepared by directly blending the traditionally dissolved P3HT molecules and CdSe NTs in DCB. All the hybrid solutions were filtered through a 0.45-μm filter before use.

### Fabrication of HBSCs

Patterned ITO-coated glass substrates were cleaned sequentially with soap water, deionized water, acetone, and isopropanol under ultrasonication for 20 min. Substrates were then dried under N_2_ flow after which a compact TiO_2_ layer was deposited on top by spin-coating a titanium-acetylacetone precursor and then sintering at 450 °C for 90 min. The ITO/TiO_2_ substrates were transferred into a N_2_-filled glovebox where the fully mixed P3HT:CdSe NTs or P3HT NWs:CdSe NTs DCB solution was spin-coated. The freshly prepared hybrid films underwent solvent treatment with 1,2-ethanedithiol (EDT):methanol (10% by volume) before annealing at 150 °C for 10 min. With regard to P3HT NWs:CdSe NTs samples, keeping them in vacuum (with pressure lower than 10^-2^ Pa) for 12 h was required prior to heat treatment. The fabrication of hybrid solar cells was finished by evaporation of 3 nm MoO_3_ followed by 100 nm Ag on top. A shadow mask was used to define six separated devices each with a diameter of 2 mm.

### Measurements

The morphology of synthesized P3HT NWs and CdSe NTs was confirmed by high-resolution transmission electron microscope (HR-TEM, JEM-2100) at an acceleration voltage of 200 kV. The crystal structure was researched by X-ray diffraction (XRD) on a Rigaku D/max-gA X-ray diffractometer with Cu Kα radiation. Light absorption measurements were carried out on Varian Cary-5000 model Ultraviolet-visible infrared spectrophotometer. Photoluminescence (PL) spectra were collected on HORIBA Jobin Yvon Fluorlog-3 system, with exciting wavelength of 360 nm. Time-resolved photoluminescence (TRPL) spectroscopy measurements (FLSP920 lifetime spectrometers, Edinburgh Instruments, EI) were conducted using a pulse laser (380 nm) with a pulse width of 70 ps for excitation. The current-voltage characterizations were carried out using a Keithley 2440 source meter and Newport 94043A solar simulator (AM 1.5 illumination). For the surface photovoltage (SPV) spectra measurements, the samples were excited with a laser radiation pulse (wavelength of 355 nm and pulse width of 5 ns) from a third-harmonic Nd:YAG laser (Polaris II, New Wave Research, Inc.)

## Results and Discussion

**Fig. 1:**
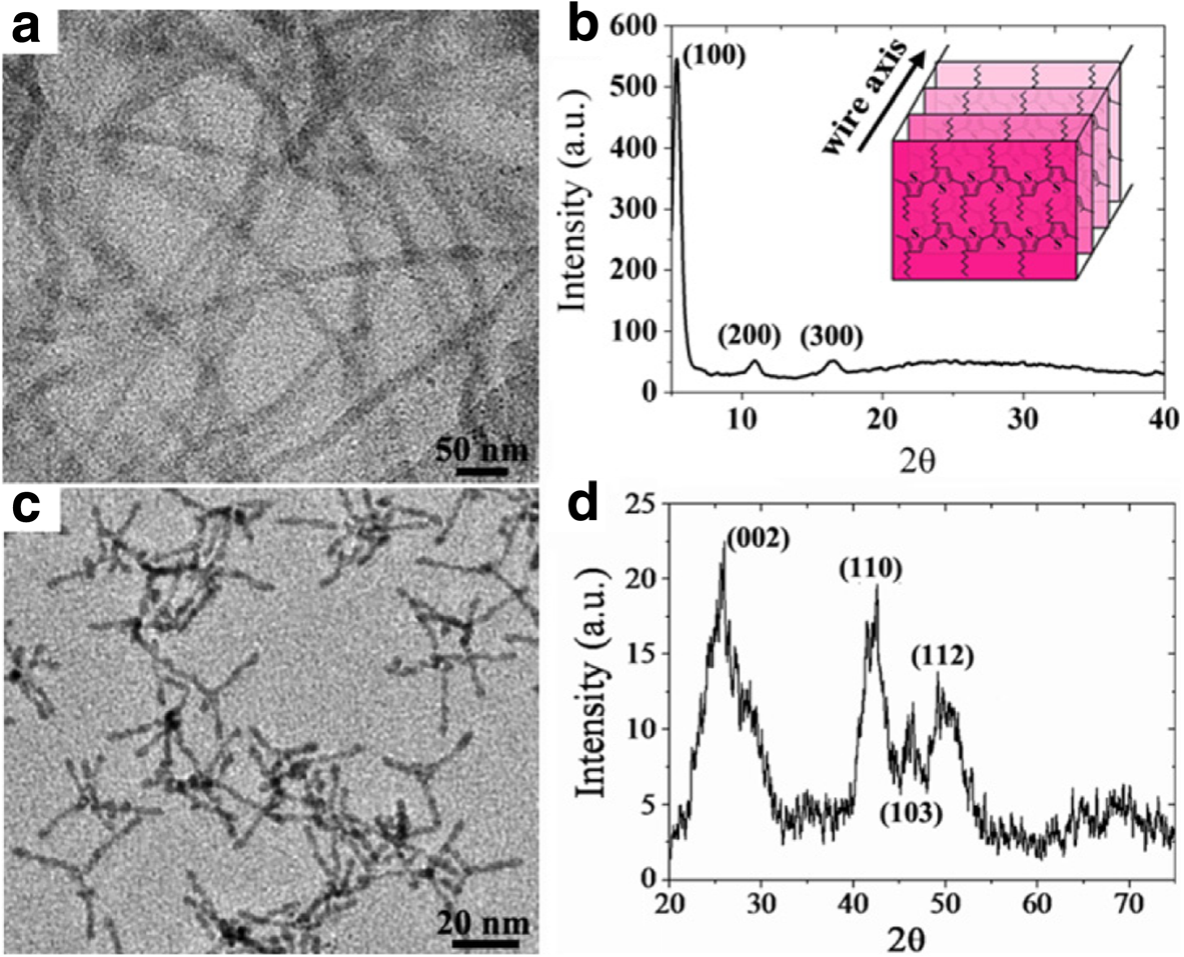
TEM image (**a)** and (**b**) XRD result of synthesized P3HT NWs, TEM image (**c**) and XRD (**d**) result of CdSe nanotetrapod. *Inset* in (**b**) is the skeleton of self-assembled P3HT molecules

The TEM image of synthesized P3HT NWs is shown in Fig. 1a. As is seen, the NWs show a uniform diameter of about 15 nm. They have a good monodispersion in organic solvent such as chlorobenzene and dichlorobenzene, which is beneficial to the formation of hybrid films. Figure 1b shows the XRD results of synthesized P3HT NWs. The three diffraction peaks can be assigned to the (100), (200), and (300) planes [25]. The first intense peak at about 5.3 ° indicates the P3HT molecules packs at the (100) plane and epitaxial growth toward [100] direction, as is depicted in the inset of Fig. 1a. Figure 1c shows the morphology of CdSe NTs. The NTs have an average arm length and diameter of about 20–25 nm and 4–5 nm, respectively. The good crystallization of CdSe NTs is demonstrated by XRD characterization that confirms a wurtzite phase (Fig. 1d).

**Fig. 2:**
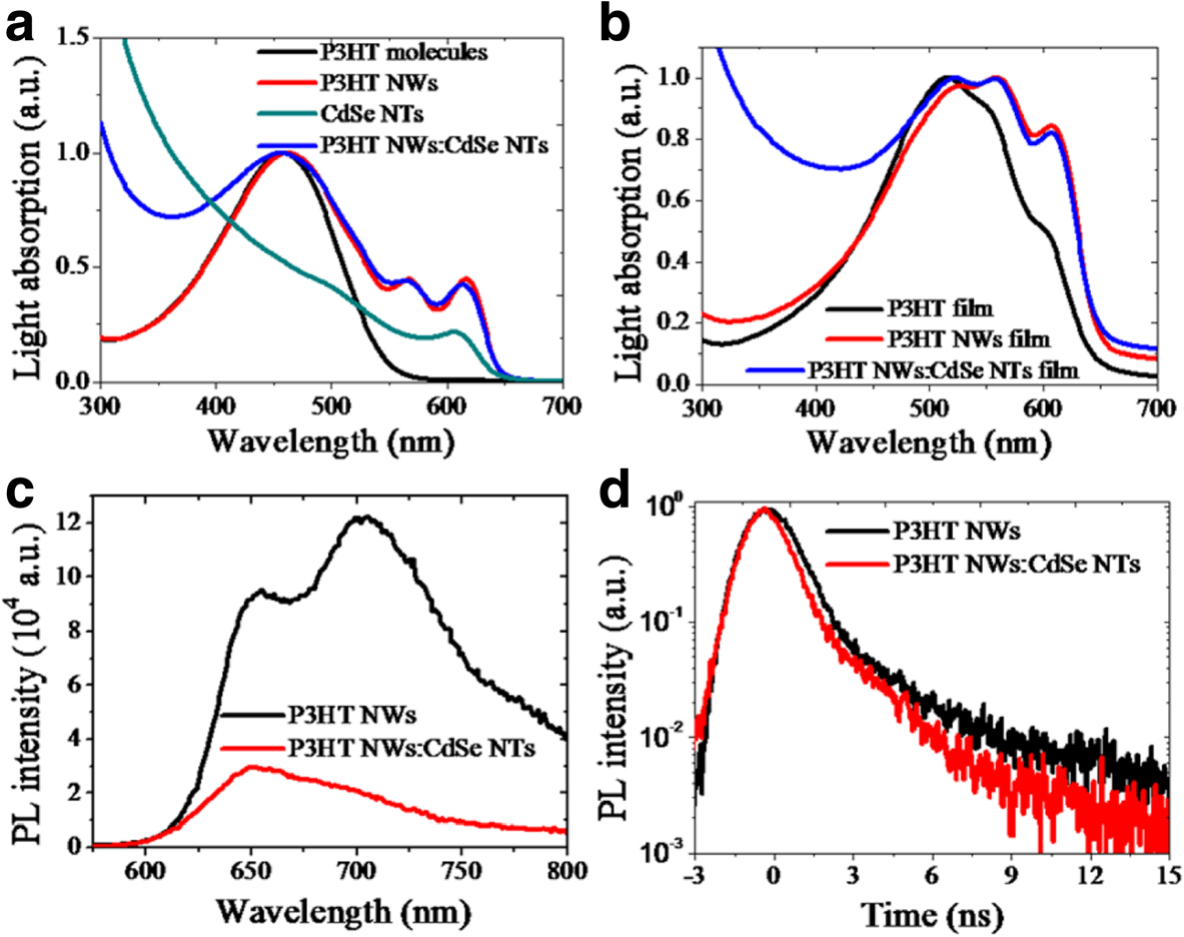
Normalized light absorption properties of different samples in (**a**) solution and (**b**) thin films, (**c**) steady state, and (**d**) time-resolved photoluminescence properties of P3HT NWs and the hybrids.

Light absorption properties were characterized and the results are shown in Fig. 2. The absorption of P3HT molecules in DCB was also measured, which shows a broad absorption peak with the light response edge at about 550 nm (Fig. 2a). Comparatively, the P3HT NWs shows a much broadened absorption curve with the response edge greatly red-shifted to about 650 nm. Typically, two distinct peaks at 560 and 610 nm appear, indicating that the monodispersed P3HT NWs sample in DCB is highly crystallized through π-π stacking [26, 27]. When blended with CdSe NTs, the hybrid mixture in DCB has an intensely enhanced absorbance at short wavelength because of the characteristic light response of CdSe NTs. For solar cells application, the absorption of different thin-film samples were also tested (Fig. 2b). The P3HT film prepared by spin-coating P3HT molecules and thereafter annealing exhibits the typical two weak shoulder peaks at about 560 and 610 nm, which demonstrates a regioregular stacking of P3HT molecules in the traditionally annealed film [28, 29]. It is noted that the absorption intensity at these two vibronic peaks for P3HT NWs film is greatly enhanced compared to the main peak at 520 nm which is red-shifted from that observed in the solution samples due to the stronger intermolecule interaction. This also means the P3HT NWs film has a much enhanced crystallization phase that helps to increase light absorption at long wavelength. Similarly, the hybrid film sample composed of P3HT NWs and CdSe NTs shows greatly broadened and strong light response from 300 to 650 nm, which will benefit the photovoltaic performance of hybrid solar cells.

To investigate the exciton-splitting property in the hybrid containing the two nano-building blocks, we carried out photoluminescence characterization and the results are shown in Fig. 2c. The PL spectra for P3HT NWs exhibits two distinct emission peaks at 660 and 700 nm, corresponding to the two excitation absorption at 520 and 560 nm (Fig. 2b), respectively. It is found that a hybrid containing 80 wt% CdSe NTs could intensively quench the PL intensity of P3HT NWs, especially the main emission at 700 nm. This is interestingly the same with that of the organic polymer-grafted nanoparticles reported in ref [30]. The static PL result indicates that the photon-generated excitons could be efficiently dissociated through diffusing in the crystallized P3HT NWs and thereafter splitting at the hybrid interface. To give additional information on the charge transfer dynamics, time-resolved photoluminescence measurement was carried out as shown in Fig. 2d. The PL lifetime analyzed by biexponential decay kinetics is 454 ps for solid state P3HT NWs, while it is 345 ps for the P3HT NWs:CdSe NTs hybrids. The PL decay of the hybrid nanostructures is much faster than that of the homopolymer, demonstrating a rapid charge transfer from P3HT NWs to CdSe NTs before radiated recombination.

**Fig. 3:**
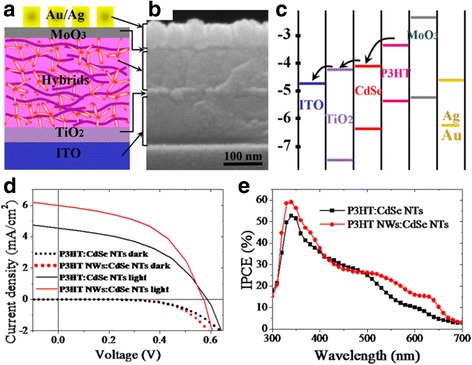
The device skeleton (**a)**, cross-sectional SEM image (**b**), and energy level alignment of the hybrid solar cell (**c**). J-V charactgeristics (**d**) and EQE properties (**e**) of the solar cells with traditional P3HT molecule and P3HT NWs as the electrons donor. Averaged data from six samples for each hybrid structure were used here. The performance of P3HT:CdSe NTs (with 1:6 mass ratio) reference solar cell was optimized

The photovoltaic device containing P3HT NWs and CdSe NTs hybrids as the active layer was fabricated with an architecture: ITO/TiO_2_/P3HT NWs:CdSe NTs/MoO_3_/Au/Ag, as shown in Fig. 3a. Blending the two nano-building blocks will construct a cross-linked network with interpenetrated and bicontinuous channels for extraction of electrons (through CdSe NTs) and holes (through P3HT NWs). The cross-sectional SEM image of the obtained device is given in Fig. 3b, which clearly exhibits a multilayered structure. A dense TiO_2_ layer with the thickness of 15 nm is required for electrons collection as well as holes blocking. The anode buffer layer (MoO_3_) is as thin as 3 nm that it is difficult to be observed. Figure 3c shows the energy level alignment of the entire solar cell. As can be seen, the cascade type-II heterojunction of MoO_3_/P3HT:CdSe/TiO_2_ theoretically guarantees an efficient charge transfer and collection after excitons dissociation at P3HT NWs:CdSe NTs interface.

Figure 3d shows the I-V characteristic of the hybrid solar cell with P3HT NWs as electron donor. For comparison, the solar cell adopting traditional P3HT molecules as donor was also fabricated and optimized as reference (named P3HT:CdSe NTs); its photovoltaic performance was also given in Fig. 3d. Under AM 1.5 illumination, the reference solar cell with traditional P3HT molecules donor generates a short current density (Jsc) of 4.5 mA/cm^2^, open circuit voltage (Voc) of 0.58 V, fill factor (FF) of 45.9% and conversion efficiency (Eff) of 1.2%. In comparison, hybrid solar cell incorporating P3HT NWs donor generates much better photovoltaic performance than the P3HT:CdSe NTs device, with Jsc, Voc, FF, and Eff values of 6.0 mA/cm^2^, 0.57 V, 49.7 and 1.7%, respectively. It should be noted that the above optimized I-V performance was obtained at an active layer thickness of 150 nm for the P3HT NWs:CdSe NTs device and 120 nm for the reference. Too thick absorber layer always decreases the performance due to much enlarged series resistance. As the P3HT NWs:CdSe NTs device performs better at a relatively thick active layer, the light absorption and charge transport ability in this bicontinuous P3HT NWs:CdSe NTs hybrid is probably higher than that in the P3HT:CdSe NTs. This also could be reflected from the observation that the performance improvement is mainly benefited from the greatly enlarged Jsc and FF values those are speculated to be closely correlated with light absorption and charge carriers transport properties of the devices [31, 32]. For further comparison, external quantum efficiency (EQE) was measured and the results are shown in Fig. 3e. EQE enhancement is observed at the wavelength from 550 to 650 nm and wavelength below 400 nm. As indicated in Fig. 2b, compared to P3HT molecules, the highly crystallized P3HT NWs have much stronger absorption at the long wavelength from 550 to 650 nm, which could partly explain the EQE enhancement at this region and the increased Jsc of the solar cell.

**Fig. 4:**
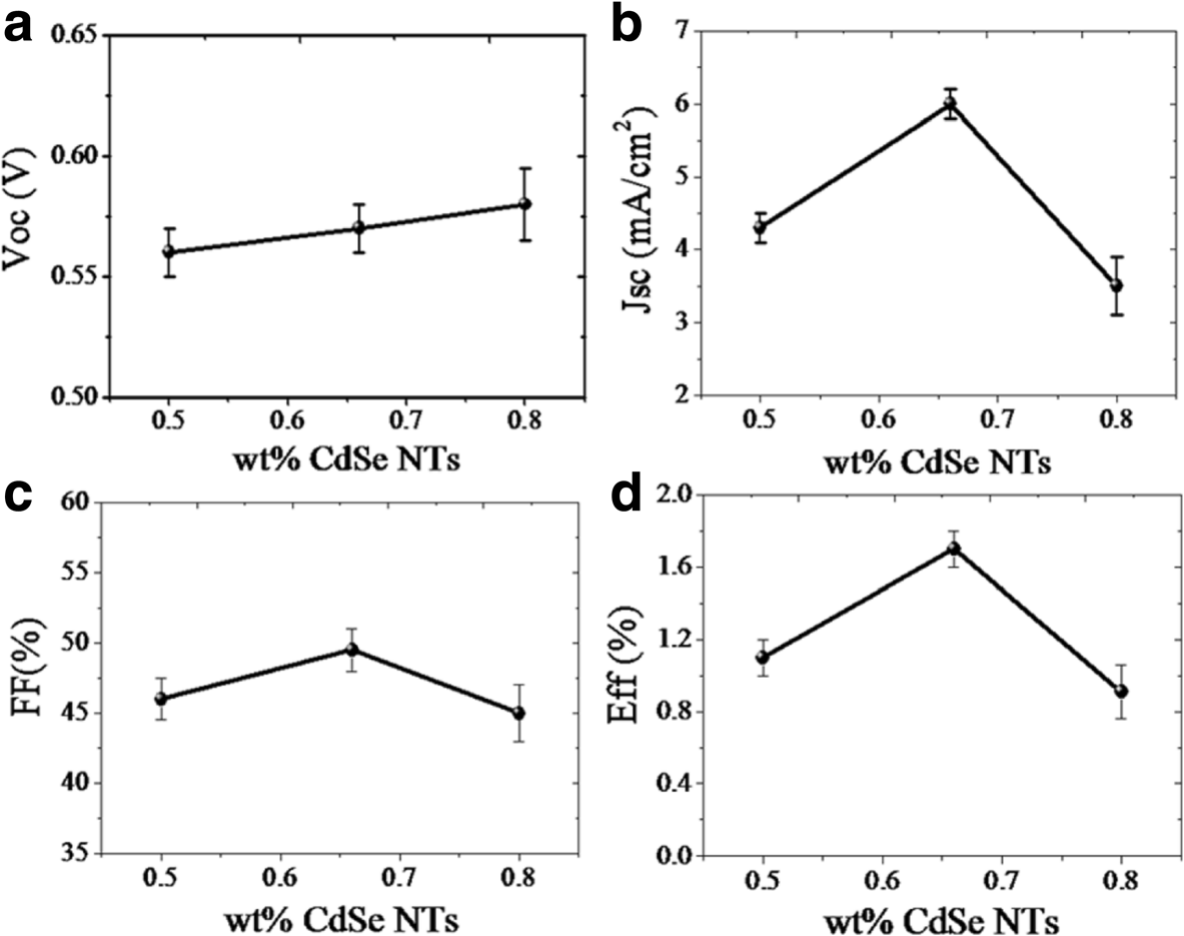
P3HT NWs:CdSe NTs mass ratio dependence of photovoltaic performance (**a**) Voc, (**b**) Jsc, (**c**) FF, and (**d**) Eff. The statistical data were from six devices for each mass ratio

The performance of hybrid solar cells adopting P3HT NWs and CdSe NTs was found to be greatly dependent on the mass ratio of electrons donor and acceptor. As shown in Fig. 4, the Voc value slightly increases with increasing the mass ratio of CdSe NTs in the hybrid while the value reaches a maximum for Jsc and FF at the 1:2 mass ratio of P3HT NWs:CdSe NTs. The obtained variation of conversion efficiency was thus found to follow the trend of Jsc and FF. Besides the light absorption, the improved charge transport is also speculated to be one of the reasons for performance enhancement of the solar cells adopting P3HT NWs as donor, which could be achieved at proper NWs/NTs mass ratio that enables interpenetrated donor/acceptor networks. It is noted that the optimized P3HT:CdSe mass ratio in our work (1:2) using P3HT NWs as electron donor is much larger than that in the reference solar cell (1:6) and the reported solar cell both with P3HT molecules as the donor [21].

**Fig. 5:**
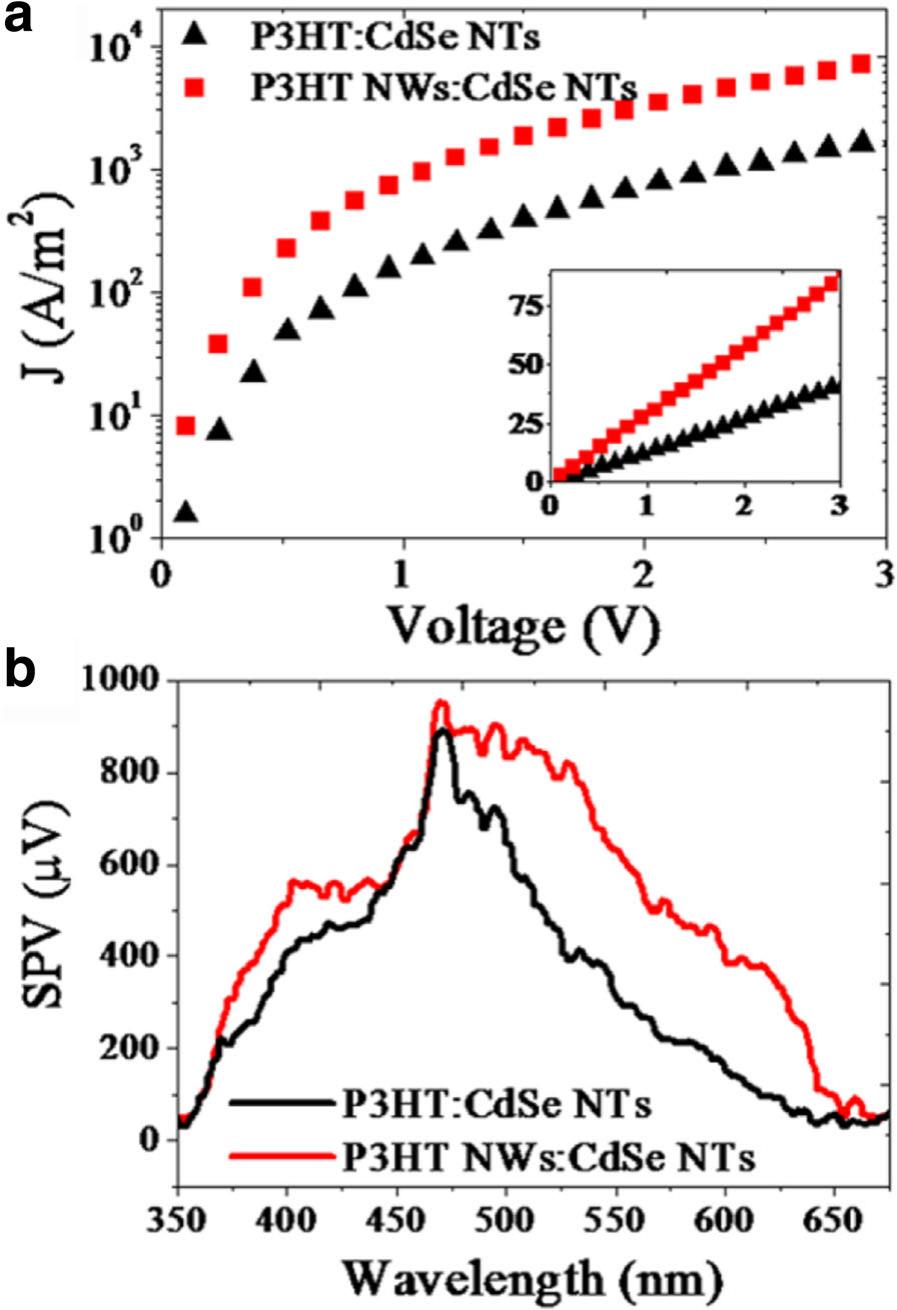
**a** Dark-current density-voltage curves for P3HT:CdSe NTs and P3HT NWs:CdSe NTs hole-only devices. The *inset* shows a linear relationship between J^1/2^ and V. **b** Surface-photovoltage spectra of the two solar cells

As mentioned above, the charge transport property should make a significant influence on the performance of two kinds of solar cells. To evaluate this property in the hybrid blends containing P3HT NWs or P3HT molecules, hole-only devices were fabricated and the space-charge limited current (SCLC) was analyzed [33]. Figure 5a shows the relationship between dark-current density (J) and voltage (V) in the hole-only devices with structure of ITO/PEDOT:PSS/P3HT NWs (or P3HT molecules):CdSe NTs/MoO_3_/Au. The J-V data were analyzed by using the nonlinear least-squares fitting to the modified Mott-Gurney equation (Eq. 1),

1 $$ J=\frac{9}{8}\varepsilon {\varepsilon}_0\mu \frac{V^2}{L^3} \exp \left(\frac{0.89\beta }{\sqrt{L}}\sqrt{V}\right) $$

where *J* is the current density, *V* is the applied voltage, *L* is the thickness of active layer, *μ* is the mobility, *ε* is the relative permittivity, *ε*_*0*_ is the permittivity of free space, and *β* is the field-activation factor [33]. The SCLC hole mobility in the optimized P3HT NWs:CdSe NTs hybrid (optimized thickness 150 nm) is calculated to be 7.9 × 10^-4^ cm^2^/Vs while it is 9.2 × 10^-5^ cm^2^/Vs in the annealed P3HT:CdSe NTs reference (optimized thickness 120 nm). The obviously increased hole mobility indicates that the P3HT NWs:CdSe NTs device has a more efficient charge transport and collection ability compared to the reference device. The increased charge motility could also be demonstrated by surface photovoltage spectra shown in Fig. 5b. As is seen, through 370 to 650 nm, the SPV signal of P3HT NWs:CdSe NTs hybrid device produces an obviously enlarged SPV signals compared to that of the P3HT:CdSe NTs reference, indicative of an increased number of charges that can transport to the hybrid film surface after exciton dissociation [34, 35].

**Fig. 6:**
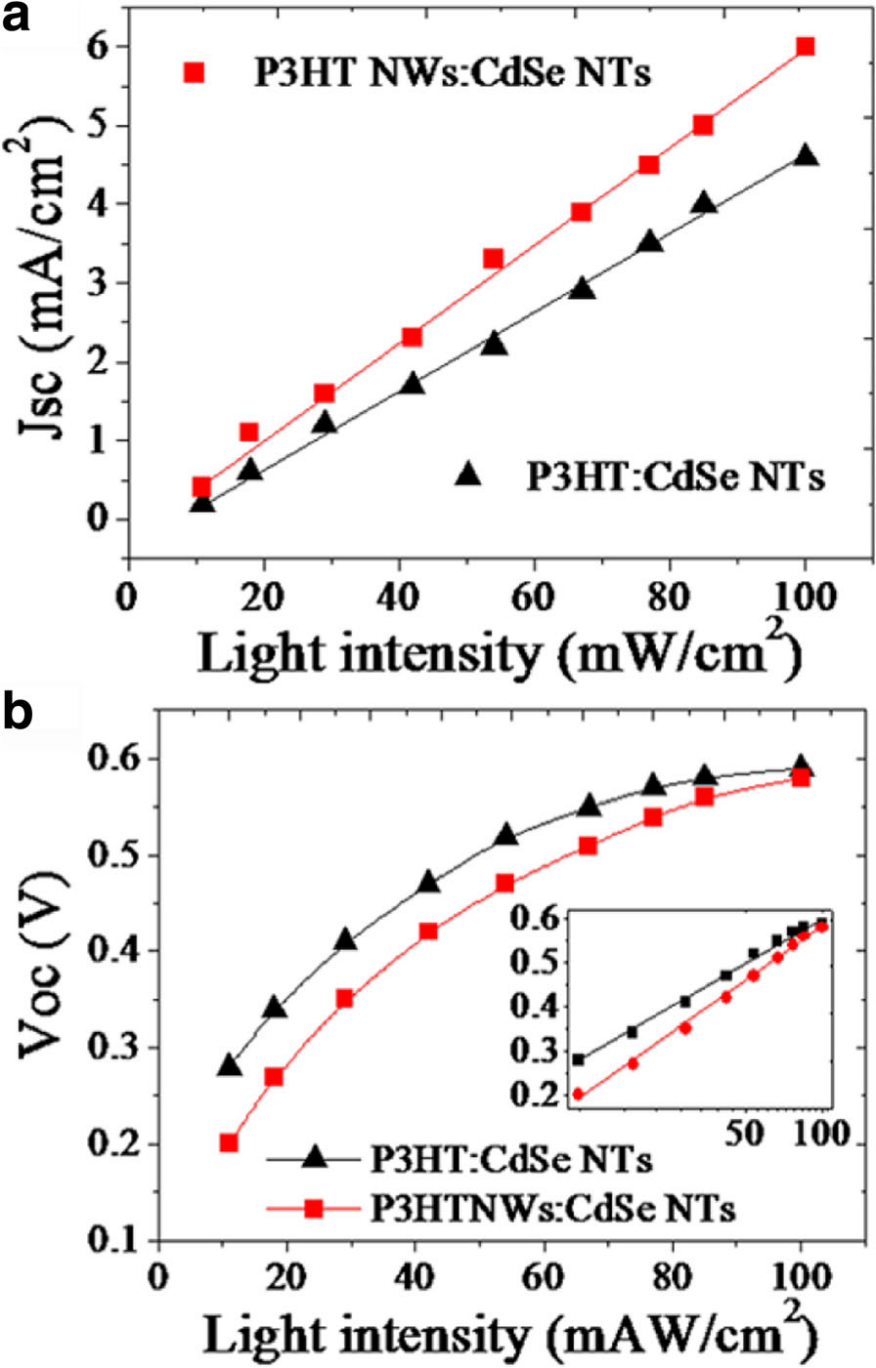
Light intensity dependence of (**a**) Jsc and (**b**) Voc in different cells. *Inset* in (**b**) shows a logarithmic increase of Voc

For further validation, light intensity dependence of Jsc and Voc is researched to evaluate the efficiency of charge collection. Shown in Fig. 6a is that both of the two solar cells present near-linear variation of Jsc following the increase in light intensity. In comparison, Jsc value increases more rapidly in the P3HT NWs:CdSe NTs solar cell than that in the P3HT:CdSe NTs reference cell, demonstrating an enhanced charge collection in the former device at high light intensity [36]. Figure 6b also shows the similar logarithmic increase of Voc with light intensity in the two hybrid solar cells. Both of two devices have the maximum value of Voc at 100 mW/cm^2^, 0.58 and 0.59 V for the P3HT:CdSe NTs and P3HT NWs:CdSe NTs, respectively. The results indicate that charge collection and extraction is more effective in the P3HT NWs:CdSe NTs hybrid than that in the P3HT:CdSe NTs hybrid. Accordingly, I-V performance is benefited from the enhanced charge collection in the P3HT NWs:CdSe NTs solar cell.

## Conclusions

In conclusion, we have fabricated organic/inorganic hybrid bulk-heterojunction solar cells integrating two efficient nano-building blocks: P3HT NWs as the electron donor and CdSe NTs as the acceptor. Broad light absorption and efficient charge transport properties were retained in this novel hybrid system. More importantly, efficient charge collection and extraction were readily acquired because of the bicontinuous charge channels in the hybrids containing these two nano-building blocks. Thus compared to the traditional P3HT:CdSe NTs hybrid, an averaged enhancement of 42% in photovoltaic performance was achieved for the solar cells adopting P3HT NWs:CdSe NTs hybrid. Our work provides a novel hybrid architecture for efficient bulk-heterojunction optoelectronic devices.
